# Cortical and subcortical gray matter structural alterations in normoglycemic obese and type 2 diabetes patients: relationship with adiposity, glucose, and insulin

**DOI:** 10.1007/s11011-018-0223-5

**Published:** 2018-04-13

**Authors:** Gabriel Bernardes, Richard G. IJzerman, Jennifer S. ten Kulve, Frederik Barkhof, Michaela Diamant, Dick J. Veltman, Jesus Landeira-Fernandez, Liselotte van Bloemendaal, Eelco van Duinkerken

**Affiliations:** 1 Department of Psychology, Pontifíca Universidade Católica – Rio de Janeiro, Rua Marquês de São Vincente, 225, Gávea, Rio de Janeiro, RJ 22451-900 Brazil; 2 Amsterdam Diabetes Center/Department of Internal Medicine, VU University Medical Center, Amsterdam, The Netherlands; 3 Department of Radiology and Nuclear Medicine, VU University Medical Center, Amsterdam, The Netherlands; 4 Institutes of Neurology and Healthcare Engineering, University College London, London, UK; 5 Department of Psychiatry, VU University Medical Center, Amsterdam, The Netherlands; 6 Department of Medical Psychology, VU University Medical Center, Amsterdam, The Netherlands; 7 Center for Epilepsy, Instituto Estadual do Cérebro Paulo Niemeyer, Rio de Janeiro, RJ Brazil

**Keywords:** Obesity, Type 2 diabetes, Neuroimaging, Brain structure, Insulin, Glucose

## Abstract

Type 2 diabetes (T2DM) is associated with structural cortical and subcortical alterations, although it is insufficiently clear if these alterations are driven by obesity or by diabetes and its associated complications. We used FreeSurfer5.3 and FSL-FIRST to determine cortical thickness, volume and surface area, and subcortical gray matter volume in a group of 16 normoglycemic obese subjects and 28 obese T2DM patients without clinically manifest micro- and marcoangiopathy, and compared them to 31 lean normoglycemic controls. Forward regression analysis was used to determine demographic and clinical correlates of altered (sub)cortical structure. Exploratively, vertex-wise correlations between cortical structure and fasting glucose and insulin were calculated. Compared with controls, obese T2DM patients showed lower right insula thickness and lower left lateral occipital surface area (*P*_FWE_ < 0.05). Normoglycemic obese versus controls had lower thickness (*P*_FWE_ < 0.05) in the right insula and inferior frontal gyrus, and higher amygdala and thalamus volume. Thalamus volume and left paracentral surface area were also higher in this group compared with obese T2DM patients. Age, sex, BMI, fasting glucose, and cholesterol were related to these (sub)cortical alterations in the whole group (all *P* < 0.05). Insulin were related to temporal and frontal structural deficits (all *P*_FWE_ < 0.05). Parietal/occipital structural deficits may constitute early T2DM-related cerebral alterations, whereas in normoglycemic obese subjects, regions involved in emotion, appetite, satiety regulation, and inhibition were affected. Central adiposity and elevated fasting glucose may constitute risk factors.

## Introduction

Type 2 diabetes mellitus (T2DM) and obesity are both worldwide health concerns, which are related to an increased risk of cardio- and cerebrovascular disease, cancer, cognitive impairment, and dementia (Reijmer et al. 2010; McCrimmon et al. 2012; Karlin et al. 2012). Cerebral damage, such as loss of (sub)cortical gray and white matter volume, and changes in brain functioning are also frequently observed in obese and T2DM groups (Widya et al. 2011; McCrimmon et al. 2012; Moran et al. 2013; Moulton et al. 2015).

In T2DM patients, a recent meta-analysis of 23 volumetric neuroimaging studies showed reduced global brain, as well as loss of hippocampus, basal ganglia, orbitofrontal, and occipital volume (Moulton et al. 2015), and a large case-control study reported loss of brain volume in T2DM patients compared to controls in areas that are commonly atrophic in Alzheimer’s disease, such as the temporal lobe, precuneus, and anterior cingulate cortex (Moran et al. 2013). Other studies have shown deficits in cortical thickness and surface area diffusely distributed throughout the brain (Brundel et al. 2010; Peng et al. 2015). It is generally accepted that micro- and macroangiopathy, the first serving as a marker for chronic hyperglycemic exposure, constitute major risk factors for these cerebral deficits, although not all studies agree (Moran et al. 2016). Almost all of the studies so far have not used the presence of micro- or macroangiopathy as an exclusion criterion for participation, and therefore the effects of T2DM in its early stages on the brain are insufficiently clear. Ideally, in the absence of clinically manifest angiopathy it should be possible to better determine the effects of obesity, blood pressure, and disturbances in glucose and insulin metabolism/production on the brain.

The effect of obesity on cortical structure is less clear-cut. Studies in the general population have found that increased BMI is related to thinning of the left lateral occipital and right ventromedial prefrontal cortex (Medic et al. 2016), and larger waist-hip-ratio and waist circumference to total brain and global gray matter volume (Debette et al. 2014). In the latter study, higher BMI was related to increased white matter hyperintensity volume. In contrast, higher visceral fat indices were found to relate to cortical thickening in both adolescents (Saute et al. 2016) and adults (Kaur et al. 2015). Studies directly comparing obese versus non-obese participants have shown increased cortical thickness (Ronan et al. 2016), and amygdala and hippocampus volume (Widya et al. 2011). Other studies, however have shown lower cortical thickness and volume diffusely distributed throughout the brain in elderly (Brooks et al. 2013; Marqués-Iturria et al. 2013). Willette and Kapogiannis (2015) reviewed 44 studies that assessed gray and white matter volume in obesity, concluding that there may be an association between obesity and frontal gray matter atrophy across all ages, as well as temporal and parietal gray matter atrophy in middle and old age (Willette & Kapogiannis, 2015). These different results could be the result of different processes. The first possible explanation is what is called the ‘obesity-paradox’, stating that midlife obesity, but not obesity in elderly, is especially harmful for the brain. A recent meta-analysis on dementia diagnosis by Kivimäky et al. demonstrated this ‘obesity paradox’. The hazard ratio for dementia per 5-points BMI increase was below 1 for those groups measured 10 and 10–20 years before diagnosis of dementia, but was 1.16 for those measured > 20 years before dementia diagnosis (Kivimäki et al. 2017). This indicates that midlife obesity was related to an increased dementia risk, but that later life obesity resulted in lower risk of dementia. A similar effect is observed in T2DM, where midlife onset T2DM is related to (more severe) cognitive dysfunction than later-life onset T2DM (Ryan et al. 2016). This ‘reverse epidemiology’, may be driven by the shorter time of late-life obesity/T2DM to exert their negative effects on the brain, compared with midlife obesity/T2DM. Alternatively***,*** obese people are sometimes healthy, without blood pressure, lipid or glucose metabolism problems, even though they possess several risk factors for major diseases, especially regarding cardiovascular conditions (Kim et al., 2016). The differences may also be driven by various methodological differences, such as use of different variables, i.e. visceral fat or BMI, or the inclusion of obese subjects with and without glucose disorders. Due to these diverging results, it remains difficult to determine the risk factors of cerebral damage in obesity. Risk factors identified so far include BMI, hypertension, increased glucose levels, insulin resistance and cholesterol (Kullmann et al. 2016; Wennberg et al. 2016).

As it is less clear if the alterations in T2DM are driven by obesity or by diabetes and its associated complications, we aimed to perform a detailed evaluation of cortical and subcortical gray matter structural alterations in normoglycemic obese subjects, obese T2DM patients without clinically manifest micro- or macroangiopathy, and matched lean normoglycemic controls. It was hypothesized that structural alterations would be present in obesity, but more aggravated in T2DM patients. As T2DM is most prevalent during middle-age/old age, and because T2DM related comorbidities during this period are related to cognitive problems and dementia later in life (Ryan et al. 2016), patients during midlife/older age were included. Exploratively, the correlation between glucose and insulin and (sub)cortical structure was determined.

## Materials and methods

### Participants

This study was conducted in accordance with the Declaration of Helsinki and was approved by the Medical Ethics Committee of the VU University Medical Center. Written informed consent was given by all participants. In the current study, data was pooled from 2 different studies performed at the VU University Medical Center. The first study assessed the acute effects of GLP-1 infusion on reward and satiety centers in the brain (NCT01281228) (van Bloemendaal et al. 2014), from which we included 14 controls, 16 obese participants and 13 obese T2DM patients. From the other study, assessing the long-term effects of GLP-1 treatment on reward and satiety centers in the brain (NCT01363609) (ten Kulve et al. 2016), 17 controls and 15 overweight/obese T2DM patients were included.

Inclusion criteria were age between 40 and 70 years, Caucasian ancestry, right-handedness, and stable body weight (i.e. <5% change during the 3 previous months). Women had to be post-menopausal, BMI had to be >30 kg/m^2^ for normoglycemic obese, >25 kg/m^2^ for overweight/obese T2DM participants and < 25 kg/m^2^ for controls. Controls and obese participants had to be normoglycemic (i.e. fasting plasma glucose <5.6 mmol/l and 2-h glucose <7.8 following a 75 g oral tolerance test), HbA1c of obese T2DM patients had to be between 42 and 69 mmol/mol (6.0–8.5%), with only oral (metformin/sulphonylurea derivates) treatment.

Exclusion criteria were: history of neurological, cardiovascular, renal or liver diseases, psychiatric disorders, malignancies, substance abuse, use of centrally acting agents or oral glucocorticoids, and the inability to undergo MRI scanning.

### MRI

A 3 Tesla GE Signa HDxt scanner (General Electric, Milwaukee, Wisconsin, USA) was used for MRI scanning, using an 8-channel phased-array head coil. For this study, a T1-weighted fast spoiled gradient-echo (TR: 8.2 ms, TE: 3.2 ms, 1 mm slice thickness) and a T2-based Fluid Attenuating Inverse Recovery (3D-FLAIR; TR: 8000 ms, TE: 126 ms, slice thickness 1.2 mm) sequences was used. Excessive neck signal was removed by registration of a Montreal Neurological Institute standard brain (MNI-152) to each participant’s T1-MPRAGE, thereby identifying the lower border of the brain, to increase reliability of the analyses.

### Whole-brain cortical structure analysis

To calculate cortical thickness, surface area, and volume the surface-based stream of Freesurfer 5.3 (http://surfer.nmr.mgh.harvard.edu) was used. A detailed description of the pipeline can be found elsewhere (Dale et al. 1999; Fischl and Dale 2000). In short, brain images were linearly registered to Talairach space to compute seed points, the bias field inhomogeneity was corrected, skull stripped, and white matter segmented using volumetric classification. Cutting planes derived from Talairach space were used to separate both hemispheres. An initial white matter surface was generated for each hemisphere from the results of the white matter segmentation. To find white and gray matter and pial surface, these initial surfaces were nudged into the direction of the gradient. To improve the estimation of the pial surface the 3D-FLAIR was added, as the contrast between the pial surface and dura is better on a 3D-FLAIR than on a T1-weighted image. The cortical surface of each hemisphere was automatically labeled by nonlinear surface-based registration of the Desikan-Killiany atlas, which resulted in 35 cortical parcellations per hemisphere (Fischl et al. 2004). The resulting white and pial surfaces were manually checked, and corrected if necessary.

### Subcortical volume analysis

FMRIB’s Software Library 5.0.8 (FSL; http://fsl.fmrib.ox.ac.uk/fsl/fslwiki/) FIRST was used for subcortical analyses (Patenaude et al. 2011). Further details on FSL-FIRST can be found elsewhere (Patenaude et al. 2011). In short, FSL-FIRST models the outer surface of the bilateral hippocampus, thalamus, amygdala, nucleus accumbens, caudate nucleus, globus pallidus and putamen by creating a vertex-based mesh for each image. Subsequently, each voxel within the images is assigned the label of the structure to which that voxel belongs, taking into account individual variations in surface shape of each structure, as well as the presence of neighboring structures. Next, in the participant’s native space, volume of each subcortical structure is calculated. All segmentations were manually checked, and corrected if necessary. To be able to perform group comparisons, all volumes were corrected for head size by multiplying the participant’s subcortical volume by its own V-scaling factor. The V-scaling factor is obtained from FSL-SIENAX (Smith et al. 2004) and calculated by affine registration of the T1-MPRAGE brain image to MNI-152 standard brain (Jenkinson et al. 2002). This value represents the factor with which the brain volume needs to be multiplied to normalize to MNI-152 standard brain.

### Statistical analyses

Between-group participant characteristics were analyzed using one-way ANOVA with Bonferroni correction for continuous variables, Kruskal-Wallis test for non-normally distributed variables, and *χ*^2^-test for categorical variables.

First, to assess if obesity and T2DM had an effect on global cortical structure, whole brain cortical thickness, volume and surface area were compared between the groups, correcting for age, sex, hypertension, and estimated intracranial volume. Next, local effects on thickness, volume, and surface area were analyzed using FreeSurfer 5.3 vertex-wise general linear modeling for the main effect of group, again correcting for age, sex, hypertension and estimated intracranial volume. To allow for group comparisons, thickness, surface area, and volume data were smoothed with a 10 mm full width at half maximum Gaussian kernel and transformed into fsaverage standard space, to ensure comparability between scans. Clusters were identified using a cluster-wise threshold of *P* < 0.01. Correction for multiple comparisons was performed using Monte Carlo Z simulation with 10,000 iterations. The Family Wise Error (FWE) corrected *P*-value was set a *P* < 0.05, after multiplying the *P*-value by 2 correcting for testing two hemispheres. To identify brain regions with a shared influence of BMI/obesity in both T2DM and normoglycemic obese participants, a conjunction analysis was performed (Nichols et al. 2005). Normalized for head size bilateral subcortical volume was compared between groups using a multivariate ANCOVA model, corrected for age, sex, and hypertension, applying Bonferroni correction for multiple comparisons in SPSS 20. Correlations between (sub)cortical structure and medical and demographical variables were determined using forward linear regression modeling. To increase power these correlations were calculated in the whole group, adding group allocation as confounding factor. Correlations between thickness/surface area/volume and glucose/insulin were calculated in a vertex-wise way correcting for estimated intracranial volume and group allocation.

All analyses were preformed using IBM SPSS Statistics 20 (IBM SPSS, Chicago, IL) or FreeSurfer 5.3. A *P* < 0.05 was considered to be statistically significant.

## Results

### Participant characteristics

As is shown in Table 1, obese T2DM patients had higher HbA1c, fasting glucose levels, and hypertension rates, but a more favorable lipid profile than the other groups (*P <* 0.05). Compared with controls, obese T2DM patients had higher systolic and diastolic blood pressure (*P <* 0.05), triglycerides and fasting insulin levels were higher in obese T2DM and obesity groups as compared to controls (*P* < 0.05). Manual edits (mainly editing brain voxels), were performed for 8 controls, 5 normoglycemic obese, and 10 T2MD participants (*P* = 0.729). Estimated total intracranial volume was not statistically significantly different between the groups (*P* = 0.149).

**Table 1:** Participant characteristics

	Controls	Obese	Type 2 diabetes	*P* value
Age (years)	57.08 ± 7.10 (41.03–66.92)	58.01 ± 8.39 (40.32–68.09)	60.44 ± 5.05 (50.86–70.24)	0.158
Sex, male/female (% male)	16/15 (51.6)	8/8 (50)	15/13 (53.6)	0.999
Diabetes duration (years)	–	–	8.15 ± 4.81 (1–20)	–
BMI (kg/m^2^)	22.96 ± 1.64 (20.00–25.41)	32.58 ± 2.86^a^ (29.28–39.35)	32.25 ± 4.51^a^ (26.90–42.70)	<0.001
Systolic blood pressure (mmHg)	118.32 ± 16.17 (88.67–159.00)	126.75 ± 12.08 (99.00–148.00)	135.31 ± 13.11^a^ (110.67–163.00)	<0.001
Diastolic blood pressure (mmHg)	74.60 ± 10.51 (58.00–98.67)	79.14 ± 7.73 (62.00–92.00)	81.00 ± 8.84^a^ (65.33–100.00)	0.033
Antihypertensive medication use (%)	0 (0)	3 (18.8)	17 (60.7)^a, b^	<0.001
Hypertension (%)^c^	5 (16.1)	4 (25)	18 (64.3)^a, b^	<0.001
HbA1c (%)	5.61 ± 0.36 (5.00–6.00)	5.58 ± 0.27 (5.00–6.10)	6.99 ± 1.05^a, b^ (5.70–9.00)	<0.001
HbA1c (mmol/mol)	37.24 ± 1.57 (33.00–40.00)	37.62 ± 3.03 (31.00–43.00)	53.46 ± 10.74^a, b^ (39.00–77.00)	<0.001
Total cholesterol (mmol/L)	5.43 ± 0.89 (4.00–7.00)	5.66 ± 0.89 (3.70–6.90)	4.55 ± 1.38^a, b^ (3.00–8.00)	0.002
HDL cholesterol (mmol/L)	1.91 ± 0.48 (1.00–3.00)	1.42 ± 0.43 (0.81–2.44)	1.17 ± 0.33^a^ (0.87–2.00)	<0.001
LDL cholesterol (mmol/L)	3.13 ± 0.80 (2.00–5.00)	3.45 ± 0.69 (2.10–4.60)	2.39 ± 0.92^a, b^ (1.00–5.00)	<0.001
Triglycerides (mmol/L)	0.91 ± 0.41 (0.00–2.00)	1.76 ± 1.31^a^ (0.50–5.50)	1.66 ± 1.01^a^ (0.80–5.60)	0.002
Cholesterol medication use (%)	0 (0)	1 (6.3)	19 (67.9)^a, b^	<0.001
Fasting plasma glucose (mmol/L)	4.85 ± 0.56 (4.00–5.70)	5.27 ± 0.41 (4.40–5.90)	8.50 ± 2.31^a, b^ (5.70–15.00)	<0.001
Fasting insulin (pmol/L)	37.56 ± 18.75 (16.00–109.50)	83.62 ± 51.25^a^ (42.40–243.40)	90.77 ± 35.42^a^ (40.80–157.10)	<0.001
Estimated intracranial volume (mL)	1547 ± 127.70 (1352–1768)	1505 ± 173.35 (1266–1874)	1473 ± 143.67 (1272–1884)	0.149
Manual edits (%)	8 (25.8)	5 (31.3)	10 (35.7)	0.729
Control points^d^	1	0	0	–
Brain editing^e^	7	5	10	–

Data are presented as mean with standard deviation or absolute number with percentage between parentheses. The *P*-value represents the *P*-value of the overall F-test

^a^different from controls

^b^different from obese

^c^Hypertension was defined as a systolic blood pressure of 140 mmHg or above, a diastolic blood pressure of 90 mmHg or above, or the use of antihypertensive medication

^d^Adding control point in the white matter to push the white matter segmentation forward

^e^Brain editing consisting of removing excessive skull or changing the intensity of voxels that were wrongly labeled

### Cortical gray matter structure

No significant differences were found when testing for a global effect of obesity and T2MD on mean cortical thickness, mean surface area or total grey matter volume between groups (*P >* 0.05; Table 2).

**Table 2:** Values of whole brain indices of cortical structure and subcortical volume

	Controls	Normoglycemic obese	Type 2 diabetes	*P* value^d^
Cortical thickness (mm)				
Whole brain	2.47 ± 0.08	2.43 ± 0.08	2.43 ± 0.08	0.368
Left hemisphere	2.47 ± ***0.08***	2.44 ± 0.08	2.43 ± 0.09	0.313
Right hemisphere	2.46 ± 0.09	2.43 ± 0.08	2.43 ± 0.08	0.439
Cortical surface area (mm^2^)				
Whole brain	2511.09 ± 221.00	2435.03 ± 318.07	2398.78 ± 248.28	0.951
Left hemisphere	2507.86 ± 219.64	2436.05 ± 310.31	2397.12 ± 250.98	0.973
Right hemisphere	2514.31 ± 224.07	2434.00 ± 326.67	2400.44 ± 246.41	0.909
Cortical volume (mL)				
Whole brain	465.38 ± 43.61	449.97 ± 62.22	439.65 ± 35.27	0.833
Left hemisphere	233.22 ± 22.42	225.22 ± 30.57	219.98 ± 17.20	0.743
Right hemisphere	232.17 ± 21.52	224.74 ± 31.73	219.67 ± 18.38	0.911
Subcortical volume (mL)^e^				
Bilateral amygdala	1.86 ± 0.29	2.17 ± 0.25^a^	2.01 ± 0.23	0.001
Bilateral thalamus	10.16 ± 0.72	10.66 ± 0.57^a, c^	10.04 ± 0.63^***b***^	0.010
Bilateral caudate nucleus	4.65 ± 0.47	4.61 ± 0.32	4.59 ± 0.36	0.871
Bilateral putamen	6.41 ± 0.71	6.45 ± 0.57	6.24 ± 0.58	0.833
Bilateral pallidum	2.35 ± 0.21	2.44 ± 0.15	2.35 ± 0.17	0.174
Bilateral hippocampus	5.10 ± 0.52	5.31 ± 0.39	5.17 ± 0.46	0.305
Bilateral nucleus accumbens	0.63 ± 0.13	0.61 ± 0.08	0.60 ± 0.12	0.962

Data are presented as mean with standard deviation. The *P*-value represents the overall *P*-value of the F-test

^a^different from controls

^b^different from obese

^c^different from type 2 diabetes

^d^Analyses of cortical thickness, surface area and volume are corrected for age, sex, hypertension and estimated total intracranial volume. Subcortical analyses were corrected for age, sex and hypertension only

^e^Subcortical volume was corrected for head size by multiplying the participant’s subcortical volume by its own V-scaling factor, obtained by FSL-SIENAX

**Fig. 1:**
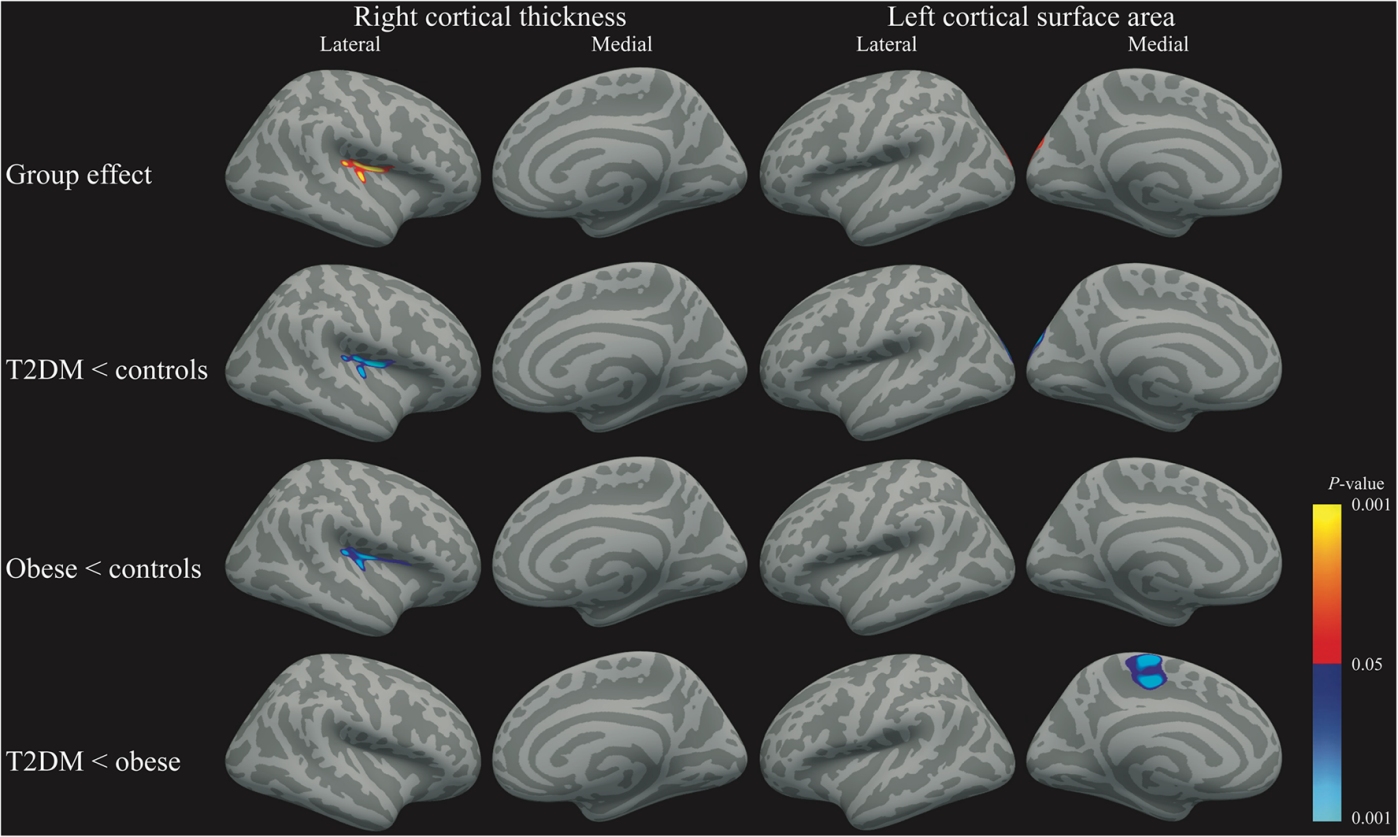
Clusters of lower cortical gray matter surface indices overlaid on a standard brain for right cortical thickness, left panel, and left surface area, right panel. Red-Yellow indicate the positive group effect, whereas blue-light blue indicates a negative effect

Corrected for age, sex, hypertension, and estimated intracranial volume there was an overall group-effect for cortical thickness in the right insula region extending into the transverse and superior temporal, supramarginal, and precentral regions (*P*_FWE_ = 0.024; Fig. 1; Table 3). For surface area, the overall group analysis showed a borderline significant cluster comprising the left lateral occipital, superior parietal and cuneus regions (*P*_FWE_ = 0.063; Fig. 1; Table 3). For cortical volume, there was no overall group-effect (all *P*_FWE_ > 0.05). Given the explorative nature of this study between-group differences for right thickness and left surface area were tested. Between-group testing showed lower cortical thickness in these regions in the T2DM group versus controls (*P*_FWE_ = 0.017; Fig. 1; Table 3). Thickness was also lower in these regions and the pars opercularis in the normoglycemic obese participants compared with the controls (*P*_FWE_ = 0.019; Fig. 1; Table 3). There were no differences between the normoglycemic obese and obese T2DM groups (*P*_FWE_ > 0.05), and the conjunction analysis showed a trend toward statistical significance for a cluster comprising the insula, extending into the transverse and superior temporal and supramarginal regions (*P*_FWE_ = 0.073). A group-effect was borderline significant for left hemisphere surface area in the lateral occipital, superior parietal and cuneus regions (*P*_FWE_ = 0.063; Fig. 1; Table 3). This effect was driven by lower surface area in T2DM patients relative to controls (*P*_FWE_ = 0.007; Figure 1; Table 3). Interestingly, T2DM patients also showed lower surface area compared with the normoglycemic obese participants, yet in the left paracentral regions (*P*_FWE_ = 0.040; Fig. 1; Table 3). There were no differences between the obese and control subjects (*P*_FWE_ > 0.05). There was no overall group-effect for cortical volume, hence no further group testing was performed (*P*_FWE_ > 0.05).

**Table 3:** Information of between-group vertex-wise analyses and whole group vertex-wise correlation analyses

	Cluster size (mm^2^)	Peak t-value	MNI coordinates peak value (x, y, z)	Anatomical location	*P*-value
Right cortical thickness					
Cluster group effect	537.38	4.347	34.0, −11.1, 16.3	Insula / transverse temporal / superior temporal / supramarginal / precentral	0.024
Cluster T2DM lower than controls	571.43	4.720	34.1, −10.3, 16.1	Insula / transverse temporal / superior temporal / supramarginal / precentral	0.017
Cluster obese lower than controls	556.05	3.380	37.9, −34.4, 12.5	Insula / transverse temporal / superior temporal / supramarginal / precentral / pars opercularis	0.019
Left surface area					
Cluster group effect	678.51	2.892	−12.7, −95.2, 20.0	Lateral occipital / superior parietal / cuneus	0.063
Cluster T2DM lower than controls	972.56	3.478	−13.3, −94.9, 20.3	Lateral occipital / superior parietal / cuneus	0.007
Cluster T2DM lower than obese	748.11	4.890	−6.9, −19.6, 67.0	Paracentral	0.040
Whole group insulin					
Cluster 1 left area negative	2008.94	−4.278	−23.6, 49.2, 9.6	Rostral middle frontal / superior frontal	0.0002
Cluster 2 left area negative	1373.58	−2.697	−64.1, −31.9, 8.5	Superior temporal / supramarginal / banks of the superior temporal sulcus	0.0008
Cluster 1 left volume negative	951.04	−2.831	−58.7, −51.3, 22.2	Superior temporal / supramarginal / banks of the superior temporal sulcus	0.001
Cluster 2 left volume negative	599.10	−3.057	−35.9, −17.2, 7.4	Insula / transverse temporal	0.020
Cluster 1 left thickness positive	491.43	4.212	−7.3, 35.2, −22.7	Medial orbitofrontal / lateral orbitofrontal	0.040

### Subcortical gray matter structure

The overall F-test including all normalized subcortical structures, corrected for age, sex, and hypertension, was statistically significant (F(2, 69) = 1.83; *P* = 0.04), indicating a difference in at least 1 subcortical structure between the groups. Post-hoc analysis showed that, after Bonferroni correction for multiple hypothesis testing, the obese group had significantly higher volume in the bilateral thalamus when compared to both other groups (all *P <* 0.03; Table 2), and in the bilateral amygdala when compared with the control group (*P =* 0.001; Table 2).

### Correlations with cortical gray matter structure

In a forward regression model, including age, sex, BMI, hypertension, waist-hip ratio, HbA1c, total cholesterol, and fasting glucose/insulin, it was tested which variables were independently related to altered gray matter structure in the whole group. We choose to do this in the whole group, because of the lack of power in the subgroups. To avoid influence of group allocation this variable was added as confounding factor.

**Fig. 2:**
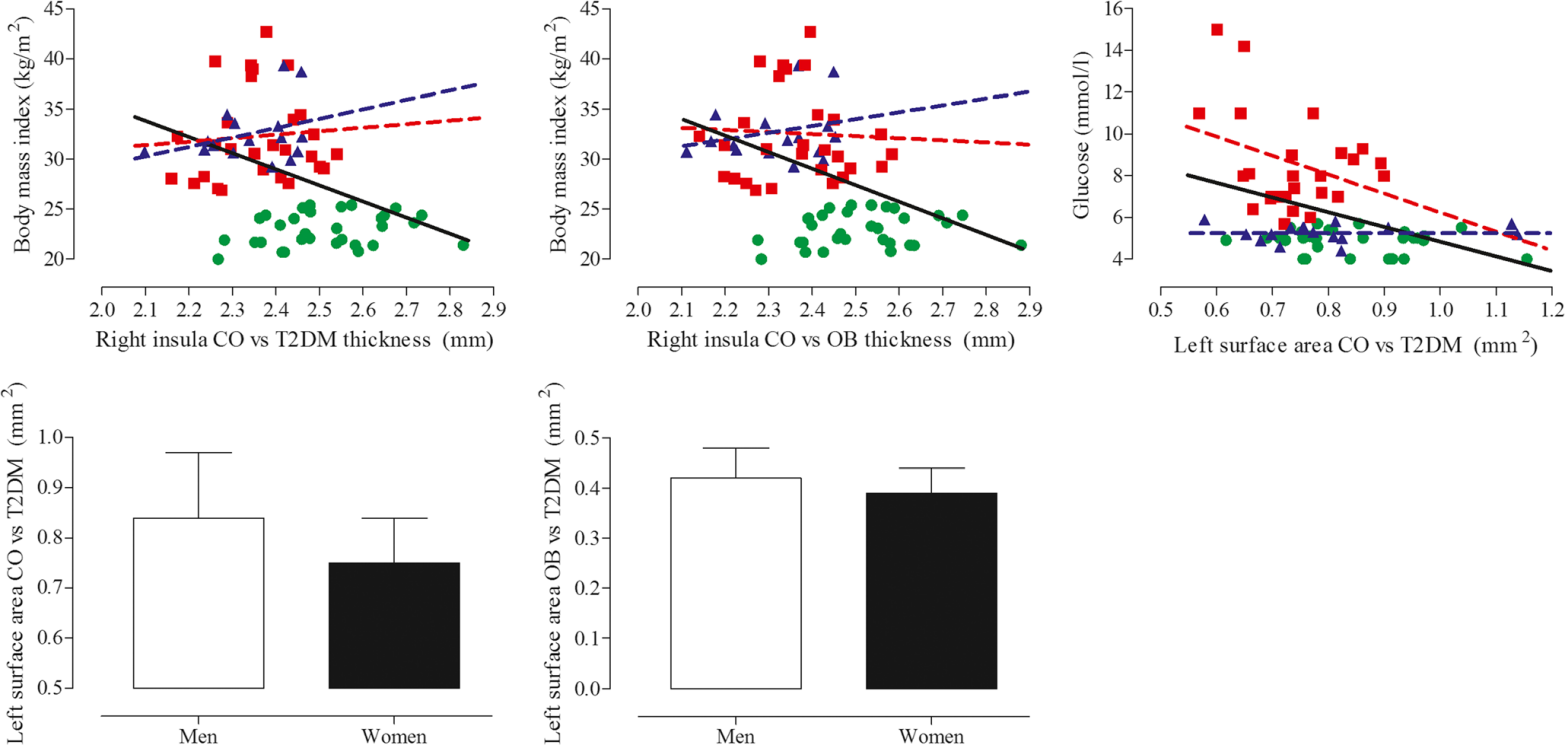
Scatter plot of the correlations between clusters of altered cortical structure and medical and anthropometric variables. Green circles depict the healthy lean controls, blue triangles the normoglycemic obese, and red squares the obese T2DM patients. The black regression line shows the correlation for the whole group. The colored regression lines depict the correlation for either the normoglycemic obese (blue) or obese T2DM (red) participants. The correlation with sex is presented as mean with standard deviation and represents men and women irrespective or group allocation

Uncorrected for group allocation there was an association between higher BMI and lower right insula thickness (cluster controls vs T2DM: β = −0.339, *P* = 0.001; cluster controls vs obesity: β = −0.431, *P* < 0.001, Fig. 2). Higher glucose (β = −0.366; *P* = 0.001) and being female (β = −0.325; *P* = 0.003) were related to lower left lateral occipital surface area. Lower left paracentral surface area was also related to being female (β = −0.238; *P* = 0.046). After correction for group allocation only the correlations between being female and lower left lateral occipital (β = −0.329; *P* = 0.003) and paracentral (β = −0.236; *P* = 0.049) surface area remained statistically significant.

### Correlations with subcortical gray matter structure

**Fig. 3:**
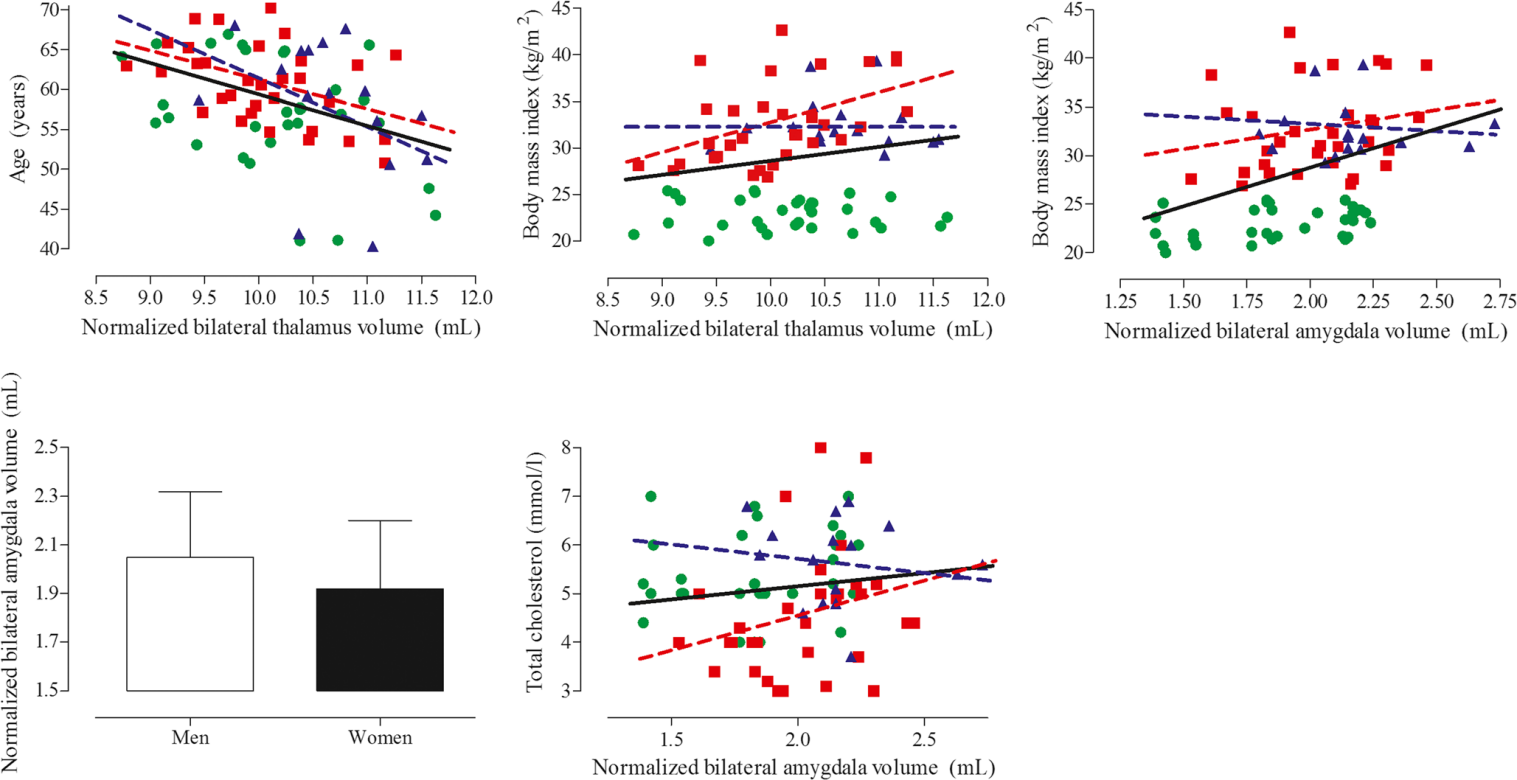
Scatter plot of the correlations between altered subcortical structures and medical and anthropometric variables. Green circles depict the healthy lean controls, blue triangles the normoglycemic obese, and red squares the obese T2DM patients. The black regression line shows the correlation for the whole group. The colored regression lines depict the correlation for either the normoglycemic obese (blue) or obese T2DM (red) participants. The correlation with sex is presented as mean with standard deviation and represents men and women irrespective or group allocation

Higher thalamus volume was related to lower age (β = −0.429; *P* < 0.001) and higher BMI (β = 0.215; *P* = 0.044; Fig. 3). Both remained statistically significantly related to thalamus volume after correction for group allocation (age: β = −0.369, *P* = 0.001; BMI: β = 0.444; *P* = 0.006). Higher amygdala volume was related to higher BMI (β = 0.495; P < 0.001), being male (β = −0.332; *P* = 0.002), and higher cholesterol (β = 0.294; *P* = 0.006; Fig. 3). Although the correlations were slightly attenuated after correction for group allocation, all remained statistically significant.

### Vertex-wise correlations with glucose and insulin

**Fig. 4:**
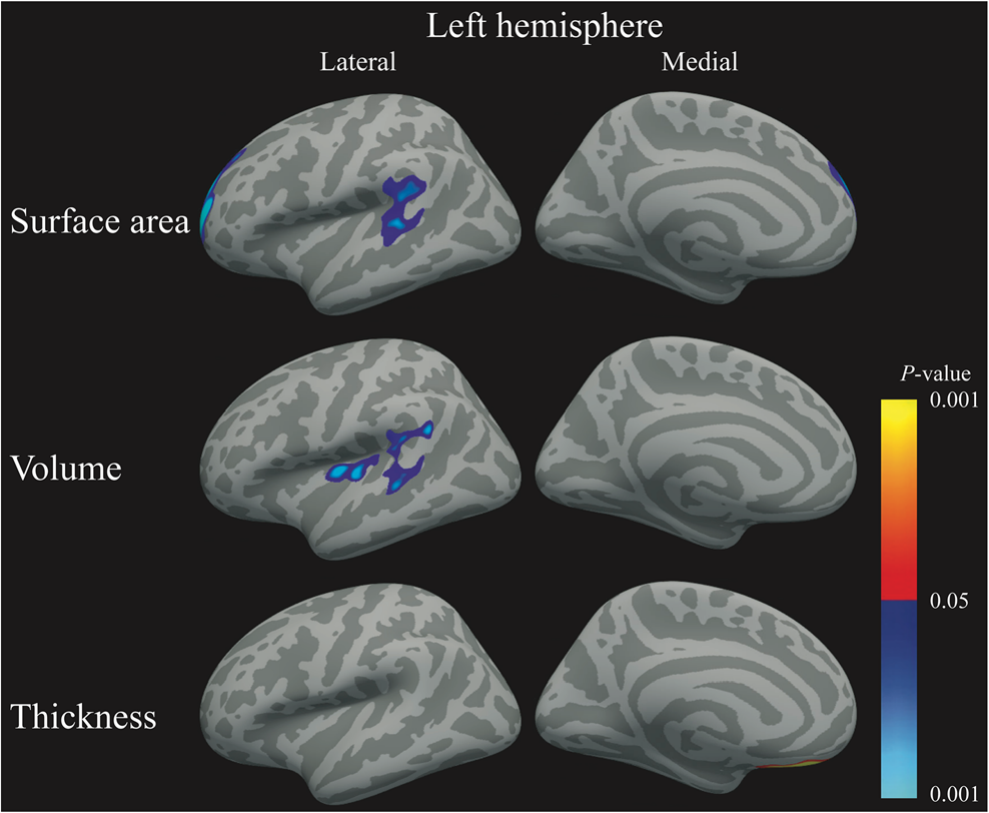
Schematic representation of the clusters where insulin, was significantly negatively related to either, surface area, thickness, or volume in all participants. Blue-light blue indicates a negative correlation, whereas red-yellow indicates a positive correlation

**Fig. 5:**
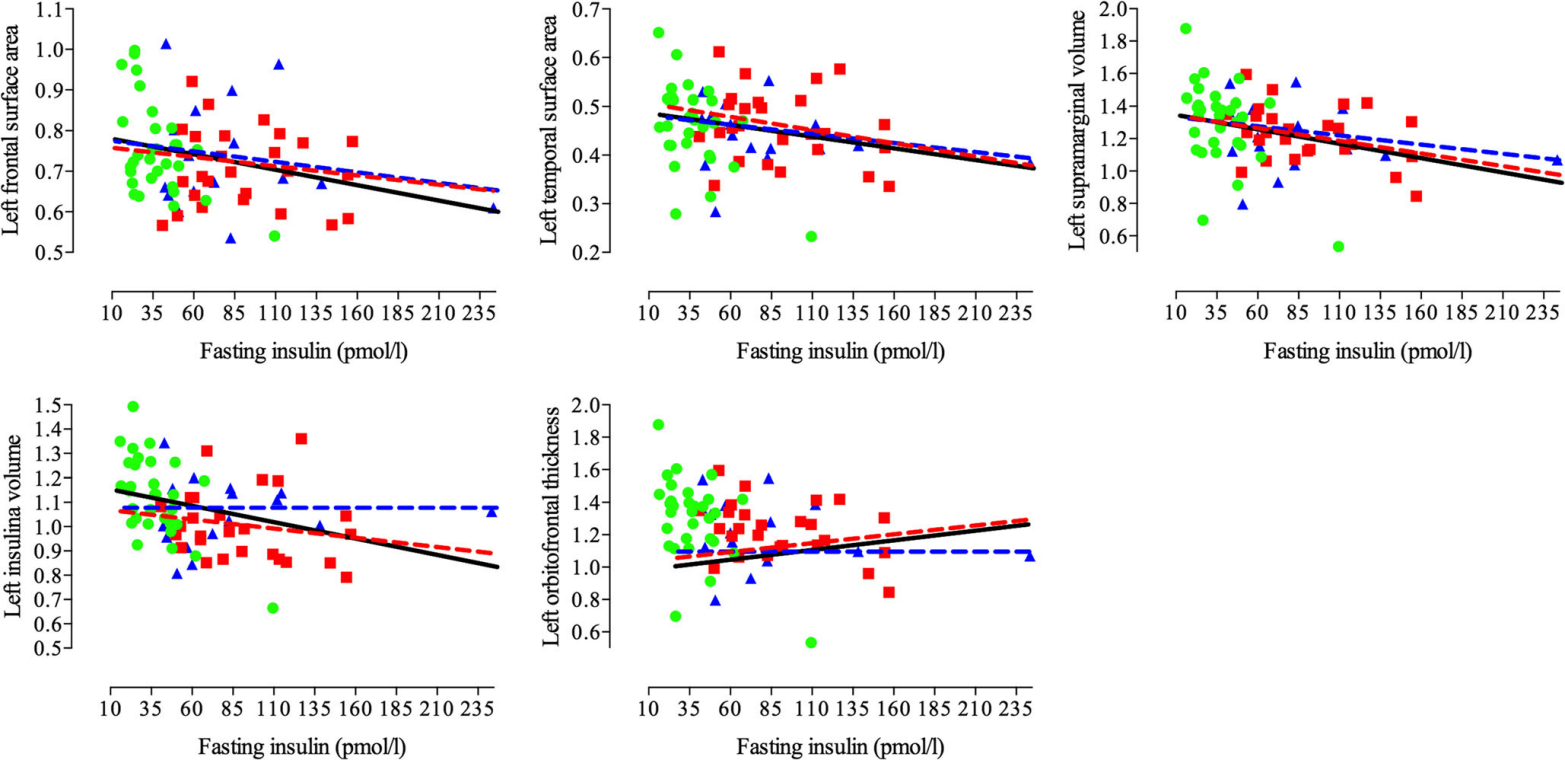
Scatter plot of the correlations between clusters that showed a vertex-wise correlation with insulin. Green circles depict the healthy lean controls, blue triangles the normoglycemic obese, and red squares the obese T2DM patients. The black regression line shows the correlation for the whole group. The colored regression lines depict the correlation for either the normoglycemic obese (blue) or obese T2DM (red) participants

All correlations are graphically presented in Fig. 4, scatter plots are shown in Fig. 5, and statistics can be found in Table 3. To increase power, these correlations were calculated in the whole group, but were corrected for group allocation and estimated intracranial volume.

Higher levels of insulin were related to lower surface area in the left rostral middle and superior frontal gyri (*P*_FWE_ < 0.001) and in a cluster comprising the left superior temporal, supramarginal, and banks of the superior temporal sulcus regions (*P*_FWE_ < 0.001). Higher insulin was also related to lower cortical volume in the left superior temporal, supramarginal, and banks of the superior temporal sulcus regions (*P*_FWE_ = 0.001) and in the left insula and transverse temporal regions (*P*_FWE_ = 0.020). Contrary, higher insulin was also related to higher cortical thickness in the left medial and lateral orbitofrontal gyri (*P*_FWE_ = 0.040). There were no correlations between insulin and the right hemisphere or with glucose and brain structure (all *P*_FWE_ > 0.05).

## Discussion

In this study, cortical and subcortical gray matter structure was studied in normoglycemic obese subjects and obese T2DM patients and compared to lean normoglycemic healthy controls. Firstly, comparing obese T2DM patients without clinically manifest micro- and macroangiopathy to controls, we saw lower right insular and temporal thickness and lower left occipital and superior parietal surface area. Secondly, comparing normoglycemic obese to normoglycemic lean control subjects, right insular, temporal and inferior frontal thickness was lower. In contrast, subcortical thalamic and amygdala gray matter volume was found to be higher. Between normoglycemic obese and obese T2DM patients, thalamic volume and left paracentral surface area were found to be lower in the latter group compared with the first. The cortical alterations were mainly related to sex, whereas the subcortical alterations were related to BMI, total cholesterol, age, and being female. Vertex-wise in the whole group, higher fasting insulin was related to lower left frontal and temporal surface area, lower temporal and insular volume, but higher orbitofrontal thickness.

The cortical structural findings of lower thickness and surface area in the temporal, parietal and occipital cortex in obese T2DM patients corroborate previous studies that also found alterations in T2DM patients in similar regions (Brundel et al. 2010; Moran et al. 2013; Moulton et al. 2015; Peng et al. 2015). Many of these previous studies, however, showed results that were spatially more widespread and found in other regions than the results from our study. An important difference with the previous studies is that in the current study no T2DM patients were included who had clinically manifest micro- or macroangiopathy. It is known that both microvascular complications and macrovascular events have a strong negative effect on brain structure in T2DM (van den Berg et al. 2009), which may explain the differences in results.

In previous obesity studies, results have been mixed; with studies showing both increased and decreased cortical and subcortical structural indices. In this study, including solely normoglycemic obese subjects, we found decreased right insular cortical thickness, which extended into the temporal and inferior frontal gyri, but no increased indices of cortical gray matter structure. On the other hand, both amygdala and thalamus gray matter volume was increased in normoglycemic obese participants. A recent study also showed decreased insula and inferior frontal gyrus volume in obese patients, thus corroborating the current results (Zhang et al. 2016). Interestingly, after bariatric surgery, there was an increased volume in the obese patients in the inferior frontal gyrus (Zhang et al. 2016). Although increased thalamus volume has not been observed previously as far as we know, higher amygdala volume has been observed in obese individuals (Widya et al. 2011). Both the amygdala and the insula are part of the limbic system and as such involved in emotion regulation. Activation due to watching food pictures in these regions has been shown to be increased in this sample of normoglycemic obese and obese T2DM patients, whereas response to actual food was decreased, linking these structures to food and satiety as well (van Bloemendaal et al. 2014; ten Kulve et al. 2016). The inferior frontal gyrus, besides involved in language processing, has a major role in response inhibition (Weywadt et al. 2016). Taking these results together, it might be hypothesized that inhibition of feeding behavior is disrupted in these obese subjects. If and how structural changes in these regions affect feeding behavior, and how and if they are involved in the pathophysiology of obesity needs to be determined in further studies.

It may be hypothesized that, as obesity is a strong risk factor for T2DM, the brain changes in T2DM are aggravated in comparison with obese subjects. In this study, in the right insula, both T2DM and normoglycemic obese participants showed lower cortical thickness, and the conjunction analysis showed a trend towards overlap within this cluster, possibly indicating that the insula is an area of overlap between both groups. Regarding cortical surface area, obese T2DM patients showed lower indices than normoglycemic obese subjects in the paracentral region. Instead of showing a continuum, both groups showed specific cortical changes in distinct regions. These diverging results may be driven by the selection of our obese participants. They had to be normoglycemic as objectified by an oral glucose tolerance test, and therefore represent a special group of obese subjects that has previously been labeled as healthy obese. The absence of impaired glucose metabolism may be driving the observed differences. It was, however, not possible to test this hypothesis as obese participants with glucose metabolism disturbances were not included in this study. Alternatively, the sample of normoglycemic obese subjects in this study was modest and lower than that of the obese T2DM and lean control participants, which may have resulted in lower power to detect alterations in other brain regions.

The structural cortical gray matter alterations found were related to being female, fasting glucose, and BMI. However, after correction for group allocation only the correlation with sex remained statistically significant. Sex is commonly known for its influence on cortical structure. Although BMI and fasting glucose were not correlated with cortical structure after correction for group allocation, it hints towards the influence of both central adiposity and glucose metabolism disturbances on the brain. Many previous studies have suggested these factors have negative consequences on the brain, but future studies should determine if they have an effect on different brain regions.

The subcortical alterations were related to age, sex, BMI, and cholesterol, independent of group allocation. Previous studies showed that cholesterol and free fatty acids were related to increased white matter integrity (Haltia et al. 2007; Verstynen et al. 2013), suggesting that abnormal lipid metabolism may relate to increased indices of cortical structure. Whereas these effects may be most profound in white matter, as the primary component of myelin is cholesterol, the results of this study show it might also be connected to amygdala volume, a deep gray matter structure that is connected and adjacent to major white matter tracts. Further studies are warranted to understand the relationship between increased brain structure and lipid metabolism.

Exploratively, the correlation between cortical gray matter structure and fasting glucose and insulin levels was determined. After correction for group allocation and estimated intracranial volume, there were no correlations with glucose. On the other hand, higher insulin was related to lower frontal, temporal, and insula surface area and volume, regions that were found affected in this study or in other obesity and T2DM studies (Moran et al. 2013; Zhang et al. 2016). Interestingly, some of these regions are part of the default mode network, a network that is highly active during rest (Greicius et al. 2004), and are considered hub regions, i.e. regions with high importance within the brain because of extensive structural and functional connections (de Haan et al. 2012). It might be hypothesized that high levels of insulin in the brain have a greater negative effect on these hub regions. This should be studied in future studies.

Limitations of this study include the relatively small sample size, especially the sample size of the normoglycemic obese subjects. Although this will have limited statistical power, the current sample size yielded sufficient power to detect between-group differences. The smaller sample size also prohibited the calculation of meaningful correlations in the groups separately. Therefore, correlations were determined in the whole group. This, however, prevents us from drawing group specific conclusions. Although the well-phenotyped normoglycemic obese and complication free obese T2DM subjects constitute a strength of the current study, it is not possible to generalize the results of this study to the general obese or T2DM population. The cross-sectional nature of this study did not allow for determination of a causal relationship. Another limitation is the difference in age range between T2DM patients and the other groups. However, this difference was small (3 years) and not statistically significant. In addition, we corrected for age in the statistical analyses. It would have been interesting to compare functional connectivity or DTI data between these participants as well, however, as this is a combined study, both fMRI and DTI data are only available for a subset of participants. Also, as the parent studies did not include cognitive testing or APOE genotyping this information is not available in this study.

In conclusion, we found insula, parietal and occipital cortical deficits in obese patients with uncomplicated T2DM, which may constitute early T2DM-related brain damage. In normoglycemic obese subjects, insula and inferior frontal gyrus deficits, but increased amygdala and thalamus volume were found, indicating a distinctly differential pattern of brain alterations in normoglycemic obese and complication free obese T2DM participants. These deficits were related to sex, but also central adiposity, and altered lipid metabolism. Lastly, insulin seems to be related to poorer cortical structural integrity in several hub regions. Future studies should focus on the pathophysiology of these cortical deficits, ultimately to ameliorate or prevent the deleterious effects of obesity and T2DM on the brain.
